# 4.4 INTERNET-BASED INTERVENTIONS FOR BIPOLAR DISORDER

**DOI:** 10.1093/schbul/sby014.011

**Published:** 2018-04-01

**Authors:** Diego Hidalgo-Mazzei, Ainoa Mateu, Maria Reinares, Francesc Colom, Eduard Vieta

**Affiliations:** 1 Hospital Clinic de Barcelona; 2 Centre for Psychiatry, Imperial College London; 3 Institute of Neuroscience, Hospital Clinic, University of Barcelona, IDIBAPS, CIBERSAM; 4 Mental Health Group, IMIM-Hospital del Mar, CIBERSAM

## Abstract

**Background:**

Adjunctive psychological interventions as an add-on to pharmacological treatment in serious mental illnesses have shown to further improve long-term outcome, especially in the case of Bipolar Disorder and first episode psychosis. Among them, psychoeducational programs have a well-established evidence of efficacy and cost-efficiency. However, there are several limitations restricting the broad implementation of these psychological treatments, out of which the most important one is related to a tremendous gap between availability and demand. Therefore, there is an emerging interest to explore new approaches to deliver this kind of treatments tailored to individual needs and in a continuous way (e.g. all year long) from any location while maintaining their efficacy at a low cost. The high availability of Internet connected devices as well as it’s user-friendly interfaces could be a potential and feasible window to expand and extend psychoeducational programs in Bipolar disorder and other serious mental illnesses. The main objective of this presentation will be: 1. to review the available internet-based psychological interventions for bipolar disorder, 2. to present the SIMPLe project development, studies protocols, results from a feasibility study and an open study, and finally, 3. to provide some insights and perspectives into the future of the field.

**Methods:**

A systematic-review of the literature was undertook to review the available internet-based psychological interventions for bipolar disorder and provide a critical appraisal of the studies and platforms included. A feasibility pilot study was conducted to test the first version of the SIMPLe app in which retention, acceptability and satisfaction were assessed in a group of subsequent samples of bipolar patients using the app, pre and post intervention questionnaires and assessments were conducted during face to face interviews. Regarding the open trial (i.e. OpenSIMPLe), a similar approach was adopted, but involving patients from all around the world and using online questionnaires.

**Results:**

During the systematic review we identified over 251 potential entries matching the search criteria and after a thorough manual review, 29 publications pertaining to 12 different projects, specifically focusing on psychological interventions for bipolar patients through diverse Internet-based methods, were selected. In the feasibility study, 51 participants were initially enrolled in the study, 36 (74%) remained actively using the application after 3 months. The whole sample interacted with the application a mean of 77 days (SD=26.2). Over 86% of the participants agreed that the experience using the application was satisfactory. So far, the OpenSIMPLe trial have enrolled more than 300 participants, preliminary results show levels of satisfaction beyond 80%, although a retention of only 5% after 6 months was calculated from servers registries.

**Discussion:**

Considering the high rates of retention and compliance reported, they represent a potential highly feasible and acceptable method of delivering this kind of interventions to bipolar patients. The results of the feasibility study confirms that this particular intervention is feasible and represent a satisfactory and acceptable instrument for the self-management of bipolar disorder as an add-on to the usual treatment but future clinical trials must still probe its efficacy. Moreover, preliminary results from the OpenSIMPLe study shows that is feasible to extend this intervention to many people at a low cost. Present and future technologies employing passive data collection and weareables could improve the personalization and accuracy of these interventions.

